# A reflection on how the absence of a psychodynamic perspective may disadvantage gender care and decision-making

**DOI:** 10.1177/10398562241312979

**Published:** 2025-01-15

**Authors:** Jillian Spencer, Roberto D’Angelo

**Affiliations:** 1974 Queensland Children’s Hospital, Brisbane, QLD, Australia; 92899 Institute of Contemporary Psychoanalysis, Los Angeles, CA, USA

**Keywords:** gender dysphoria, gender-affirming care, psychodynamic psychiatry, children, adolescents

## Abstract

**Objective:**

To reflect on factors that may have led to the widespread implementation of gender affirming care (GAC) for minors by psychiatric clinical leaders despite the absence of a robust evidence base and the known risks of harm.

**Conclusions:**

The progressive rejection of psychodynamic thinking by the profession of psychiatry may have contributed to psychiatrists failing to question key aspects of GAC for minors. Further, numerous unconscious factors potentially contribute to the foreclosure of thinking about the risks of gender medicine.

Paediatric Gender Affirming Care (GAC) is one of the most controversial issues in psychiatry and medicine today. A growing number of systematic reviews have concluded that the evidence for social transition, puberty blockade and cross sex hormones is weak.^1–5^ Importantly, there is no evidence that they reduce the risk of death by suicide as claimed.^5^ The interventions can have profound, lifelong implications, including infertility, loss of sexual function, physical health complications and regret. Furthermore, the gender affirming medical pathway is frequently followed by irreversible gender-modifying surgeries, which entail known complications and serious risks.^6,7^

Recently, the most extensive review of paediatric GAC ever undertaken was completed in the UK and led to a ban on pubertal blockade outside of clinical trials and a recommendation that cross-sex hormones only be used in very limited circumstances.^5^ For psychiatrists reflecting on the significance of these changes, the most troubling question is: how did our profession allow such poorly evidenced interventions to burgeon?

We propose that the biomedical paradigm that now dominates psychiatry, and has led to a growing rejection of psychodynamic and systemic thinking, is a significant factor. Whilst others have explored how GAC came to dominate from a service perspective^5^ or as a result of flawed research,^8^ we will be exploring how unconscious processes in individual clinicians may have been a factor.

Further, we argue that without incorporating a psychodynamic perspective, unconscious factors go unnoticed, limiting the capacity of psychiatrists to provide clinical leadership. Clinical leadership, which prevents patient harm, involves helping treating clinicians reflect on their own conscious and unconscious affective experience (countertransference) and on how it shapes their clinical stance.

Psychodynamic thinking gives clinicians a framework for thinking about patient-clinician interactions, illuminating aspects of the clinician’s and patient’s affective experience, the nature of their engagement, and other aspects of the clinical process that may not be immediately apparent. Psychodynamic thinking can shed light on a range of unacknowledged, unconscious aspects of GAC.

## GAC’s rejection of psychodynamic thinking

Perhaps the most influential factor upon psychiatrist reluctance to call for appropriate caution regarding GAC for minors is our profession’s pervasive rejection of the importance of unconscious factors. From a psychodynamic perspective, the patient’s presenting complaint and symptoms may be surface level manifestations or proxies for complex unresolved difficulties that are not in conscious awareness. This kind of thinking is no longer central to the way many psychiatrists practice. Further, when it comes to gender dysphoria, any attempts to explore the unconscious meaning and function of trans identification are mischaracterized as conversion therapy.^9^

Without a psychodynamic understanding, gender clinic formulations are likely to address only the patient’s surface level symptomatic presentation. This leads to treatment recommendations that operate at this level, namely, gender-affirming interventions, which fail to address any deeper issues. A commonly held viewpoint of gender clinicians is that children ‘know who they are’.^10^ This perspective lacks curiosity and eschews an awareness of unconscious processes. A successful therapeutic process requires a tolerance of uncertainty and the capacity to wonder about the changing internal landscape of oneself and others.

Psychodynamic theorists have devoted considerable discussion to the clinician stance in patient–clinician interactions.^11^ Contemporary psychodynamic theory has come to view the ‘blank slate’ stance that Freud recommended^12^ as an illusion. In reality, the clinician is always influencing the patient, whether s/he is aware of it or not. It is important for clinicians to reflect on how their stance and behaviour may be shaping the material that is emerging clinically. Without a psychodynamic perspective, the gender-affirming approach may appear to be benign; however, this fails to take into account the unavoidable impact that clinicians are having on their patients.

## The pride flag and the avoidance of negative affect

A striking example of GAC’s simplistic understanding of patient care is the prominence of the ‘pride flag’ in clinical settings. The Australian Standards of Care and Treatment Guidelines, which underpin practices within Australian paediatric gender clinics^13^ recommends ‘[p]roviding an environment that demonstrates inclusiveness and respect for diversity …’. To this end, LGBTQIA + advocacy organisations provide pride flags, rainbow lanyards and ally/pronoun badges that telegraph ‘respect for diversity’ to patients. As these visual symbols propagated throughout public health services, psychiatrists appear to have failed to reflect upon their clinical impact from a psychodynamic point of view.

Some might argue that these symbols simply communicate that the clinician welcomes the patient’s trans identity, and encourage a positive therapeutic alliance. This fails to consider that these visual symbols may actually shape and constrain how the clinical interaction will unfold. Rather than providing a space for understanding the child’s current struggles, relational and social context and developmental history, the consultation is being reshaped as a place to declare and confirm a transgender identity. Consequently, the child may inhibit any expression of doubts and anxieties about trans identification, or about other psychological struggles, perceiving this might disappoint the clinician. This is particularly likely if the child has a tendency to accommodate the views of others which can be a common feature of attachment disorders and responses to trauma.^14^

Such symbols may also communicate that the clinician is unable to tolerate the uncertainty that exists when a treatment alliance is being formed, further constraining the interaction. At best, the symbols will result in a positive transference and equally positive countertransference, where the narcissistic equilibrium of both is maintained, and negative interactions are avoided. At worst, the symbols may be an attempt by the clinician to control the patient to preserve the clinician’s narcissistic ideal self. The symbols may communicate that the clinician is anxious about being perceived negatively. This may convey that the clinician is unable to hold and process the patient’s own negative view of themselves.

There may be an unwillingness by the clinician to contemplate the self-loathing, body hatred, fear or trauma underpinning a patient’s stated desire to abandon their current self and re-invent themselves by changing their body. These feelings may be difficult for clinicians to tolerate and often evoke painful countertransference responses. Staying in a positive transference/countertransference relationship is therefore a collusion with the patient’s own defences against these painful affects. To remain connected to the clinician, and to the prospect of recovery, the patient must mirror the clinician by excluding or dissociating negative affect from conscious awareness and from the clinical space. This deeply distorts the therapeutic relationship, leaving the patient’s fundamental difficulties unaddressed.

## Reaction formation

Ensuring the gender clinic physical environment is decorated with trans-pride flags and related symbols is almost certainly intended to communicate that gender diversity and transition should be viewed positively by all. Any sense of alarm about the potential risks and dangers of gender-affirming interventions is replaced with an atmosphere of fun which celebrates transition.

From the perspective of psychodynamic theory, this degree of exaggeration suggests the psychic defence of reaction formation, where painful affective states are turned into their opposite.^15^ This defence not only protects the clinician from awareness of the realities and risks of gender transition: Unacceptable feelings of hostility, despair or therapeutic nihilism experienced by clinicians towards troubled and traumatised children with gender distress are expelled from conscious awareness through the performance of relentless positivity.

Clinical problems such as self-harm, body hatred and traumatic sequelae have no simple solution and require the clinician and patient to acknowledge and tolerate feelings of helplessness. At the gender clinic, helplessness is replaced with a sense of certainty that gender change will be the solution to the patient’s suffering. This is the unconscious basis of what the Cass Review described as ‘diagnostic overshadowing’ where complex psychological problems are subsumed under the diagnostic umbrella of gender dysphoria, a problem that has a singular concrete, physical solution with clearly defined steps.

## Clinician vulnerabilities

In other areas of child and adolescent psychiatry, clinicians must find ways to tolerate feelings of despair and therapeutic inadequacy. High rates of psychopathology, developmental problems, trauma and family dysfunction similarly exist in children seeking gender interventions.^16^ However, in contrast to the acknowledgement of uncertain therapeutic efficacy in other areas of child and adolescent psychiatry, the GAC pathway is marketed to clinicians as ‘quite literally lifesaving’,^17^ and ‘significantly improving’ health and wellbeing.^13^ The heroic nature of this sales message risks attracting clinicians seeking narcissistic validation.

Consequently, gender clinicians may be primed by their own psychological needs and vulnerabilities, and by unrealistic expectations derived from misrepresentations of the research evidence about the efficacy of GAC, to expect an enduring idealising transference/countertransference and patient symptom recovery from treatment. Such unrealistic expectations may render the clinician vulnerable to anxiety and defensive acting out when, inevitably, negative affect or projections enter the clinical space. For example, an unprocessed sense of persecution deriving from the patient’s negative projections, or the difficult feelings evoked in the countertransference, may evoke unconscious retaliation in the form of unreflective encouragement to pursue the gender affirming pathway with its risks and serious consequences.

Gender clinics tend to incorporate a high proportion of LGBTIQ + staff in line with valuing diversity and lived experience. However, psychodynamic concepts are especially important to hold in mind when clinicians are engaging with patients who they perceive to be experiencing similar issues to what they have experienced themselves. Stolorow termed this unacknowledged correspondence between the patient and clinician’s psychic organisation as an ‘intersubjective conjunction’ and warned that it can obscure differences between the clinician and patient, thereby preventing exploration of painful material.^18^ Gay and lesbian clinicians are likely to experience their sexual orientation as a central and immutable aspect of their identity, which may lead them to assume that their patients’ trans identity functions in the same way. Further, they may struggle with residual or unacknowledged feelings of shame related to their sexuality and may therefore avoid exploration of same-sex attraction and associated shame. A common theme in detransitioner testimonies is that many came to view their trans identification as a response to shame about same-sex attraction which was not adequately explored prior to transition.^19,20^

Recognition that a child’s stated identity may emerge from a myriad of mental health or developmental problems, or the influence of trauma or social forces, may be psychologically threatening to a gay or lesbian clinician. It may threaten a needed defensive structure that keeps the clinician’s own traumatic and developmental history at bay, particularly if this history intersects with their sexual orientation. Further, the notion that same-sex attraction is purely biological and not in any way shaped by social and relational factors, may be a pivotal construct that relieves shame. Taking anything but a gender-affirming approach, which sees gender identity as biological and immutable, may evoke significant anxiety for such clinicians.

In an act of projective identification, some clinicians may use the patient as a vessel for hated parts of the self and may subconsciously try to obtain mastery over such feelings through collusion with the patient’s fantasy of destroying their current self. Many gay adults were gender non-conforming as children. Gender non-conformity may be infused with shame for some gay and lesbian clinicians, due to experiences of bullying and shaming and may represent a hated part of the self. Authorising irreversible body-altering treatments may be an unconscious attack on the gender-non-conforming patient and a way of making these children ‘straight’ and gender-conforming, while at the same time impairing the function of the sexual body that harbours shameful same sex desires. The disturbing reality is that paediatric gender medicine may be sterilizing many young people struggling with same-sex attraction or who are yet to realise they are gay or lesbian.

Infertility seems to be a particularly poignant outcome of gender interventions for children. It is of concern that psychiatrists have not considered why adults working in gender clinics would allow distressed children to risk forgoing future deep experiences of connection derived from having biological children. An individual analysis of clinician experiences in relation to family and fertility would be required to understand the drivers behind such anti-natalism.

## Conclusion

As clinical leaders, psychiatrists authorised the implementation of paediatric gender-affirming care practices that have a weak evidence base and carry risks of significant harm. As a consequence, the profession needs to engage in an extended and open process of reckoning. Our profession’s abandonment of psychodynamic thinking may have provided the optimal environment where unconscious forces have shaped the unquestioning promotion of gender-affirming care.

## References

[1] https://cass.independent-review.uk/nice-evidence-reviews/

[2] https://archive.iftcc.org/council-for-choices-in-health-care-in-finland-palko-cohere-finland-2020-recommendation-of-the-council-for-choices-in-health-care-in-finland-palko-cohere-finland-medical-treatment-methods-for-d/

[3] https://segm.org/segm-summary-sweden-prioritizes-therapy-curbs-hormones-for-gender-dysphoric-youth

[4] https://www.floridahealth.gov/_documents/newsroom/press-releases/2022/04/20220420-gender-dysphoria-guidance.pdf

[5] Cass H . The cass review: independent review of gender identity services for children and young people: final report 2024. https://cass.independent-review.uk/home/publications/final-report/.

[6] Wang AMQ Tsang V Mankowski P , et al. Outcomes following gender affirming phalloplasty: a systematic review and meta-analysis. Sex Med Rev 2022; 10(4): 499–512. 36031521 10.1016/j.sxmr.2022.03.002

[7] Morrison SD Claes K Morris MP , et al. Principles and outcomes of gender-affirming vaginoplasty. Nat Rev Urol 2023; 20(5): 308–322. 36726039 10.1038/s41585-022-00705-y

[8] Abbruzzese E Levine SB Mason JW . The myth of “reliable research” in pediatric gender medicine: a critical evaluation of the Dutch studies-and research that has followed. J Sex Marital Ther 2023; 49(6): 673–699. 36593754 10.1080/0092623X.2022.2150346

[9] Ashley F . Interrogating gender-exploratory therapy. Perspect Psychol Sci 2023; 18(2): 472–481. 36068009 10.1177/17456916221102325 PMC10018052

[10] Minor R . Business Insider. As a gender specialist, I've seen kids as young as 3 recognize they are trans 2022. https://www.businessinsider.com/gender-specialist-trans-children-are-not-too-young-to-know-2022-1, Accessed 18 November 2024.

[11] Johnsen C Ding HT . Therapist self-disclosure: let's tackle the elephant in the room. Clin Child Psychol Psychiatry 2021; 26(2): 443–450. 33567907 10.1177/1359104521994178

[12] Freud S (1912) Recommendations to physicians practising psycho-analysis. In: *The standard edition of the complete psychological works of Sigmund Freud*. volume XII: 109-120.

[13] Telfer MM Tollit M Pace C , et al. Australian standards of care and treatment guidelines for trans and gender diverse children and adolescents version 1.4. Melbourne, Victoria: The Royal Children's Hospital, 2023.

[14] Blake-Holmes K Maynard E Brandon M . The impact of acquiescence: a model of coping developed from children of parents with mental illness. Adv Mental Health 2023; 21(3): 199–217.

[15] Baumeister RF Dale K Sommer KL . Freudian defense mechanisms and empirical findings in modern social psychology: reaction Formation, projection, displacement, undoing, isolation, sublimation, and denial. J Pers 1998; 66: 1081–1124.

[16] Kozlowska K McClure G Chudleigh C , et al. Australian children and adolescents with gender dysphoria: clinical presentations and challenges experienced by a multidisciplinary team and gender service. Human Systems 2021; 1(1): 70–95.

[17] AusPATH . Statement on NHS England interim clinical policy: “Puberty suppressing hormones for the purpose of puberty suppression for children and adolescents who have gender incongruence/dysphoria” 2024. https://auspath.org.au/2024/03/15/statement-on-nhs-england-interim-clinical-policy-puberty-suppressing-hormones-for-the-purpose-of-puberty-suppression-for-children-and-adolescents-who-have-gender-incongruence-dysphoria/, Accessed 18 November 2024.

[18] Stolorow RD Atwood GE . The intersubjective perspective. Psychoanal Rev 1996; 83(2): 181–194. 8792447

[19] Littman L O'Malley S Kerschner H , et al. Detransition and desistance among previously trans-identified young adults. Arch Sex Behav 2024; 53(1): 57–76. 38038854 10.1007/s10508-023-02716-1 PMC10794437

[20] Vandenbussche E . Detransition-related needs and support: a cross-sectional online survey. J Homosex 2022; 69(9): 1602–1620. 33929297 10.1080/00918369.2021.1919479

